# Nitrosative Stress and Nitrated Proteins in Trichloroethene-Mediated Autoimmunity

**DOI:** 10.1371/journal.pone.0098660

**Published:** 2014-06-03

**Authors:** Gangduo Wang, Jianling Wang, Xuemei Luo, G. A. Shakeel Ansari, M. Firoze Khan

**Affiliations:** 1 Department of Pathology, University of Texas Medical Branch, Galveston, Texas, United States of America; 2 Biomolecular Resource Facility, University of Texas Medical Branch, Galveston, Texas, United States of America; University of Central Florida, United States of America

## Abstract

Exposure to trichloroethene (TCE), a ubiquitous environmental contaminant, has been linked to a variety of autoimmune diseases (ADs) including SLE, scleroderma and hepatitis. Mechanisms involved in the pathogenesis of ADs are largely unknown. Earlier studies from our laboratory in MRL+/+ mice suggested the contribution of oxidative/nitrosative stress in TCE-induced autoimmunity, and N-acetylcysteine (NAC) supplementation provided protection by attenuating oxidative stress. This study was undertaken to further evaluate the contribution of nitrosative stress in TCE-mediated autoimmunity and to identify proteins susceptible to nitrosative stress. Groups of female MRL +/+ mice were given TCE, NAC or TCE + NAC for 6 weeks (TCE, 10 mmol/kg, i.p., every 4^th^ day; NAC, ∼250 mg/kg/day via drinking water). TCE exposure led to significant increases in serum anti-nuclear and anti-histone antibodies together with significant induction of iNOS and increased formation of nitrotyrosine (NT) in sera and livers. Proteomic analysis identified 14 additional nitrated proteins in the livers of TCE-treated mice. Furthermore, TCE exposure led to decreased GSH levels and increased activation of NF-κB. Remarkably, NAC supplementation not only ameliorated TCE-induced nitrosative stress as evident from decreased iNOS, NT, nitrated proteins, NF-κB p65 activation and increased GSH levels, but also the markers of autoimmunity, as evident from decreased levels of autoantibodies in the sera. These findings provide support to the role of nitrosative stress in TCE-mediated autoimmune response and identify specific nitrated proteins which could have autoimmune potential. Attenuation of TCE-induced autoimmunity in mice by NAC provides an approach for designing therapeutic strategies.

## Introduction

Autoimmune diseases (ADs) such as system lupus erythematosus (SLE), rheumatoid arthritis (RA) and scleroderma are chronic and life-threatening disorders, which contribute disproportionately to morbidity and mortality among young to middle-aged women [1], [2]. Despite relatively high prevalence of these diseases, molecular mechanisms underlying systemic autoimmune response remain largely unknown. In recent years, increasing evidence suggests the involvement of free radical-mediated reactions in the pathogenesis of ADs [3]–[12]. Indeed increased formation of reactive oxygen/nitrogen species (ROS/RNS) and oxidative/nitrosative modification of proteins are reported in various ADs [5], [9]–[11], [13], [14]. Moreover, elevated RNS/ROS-modified proteins, such as nitrotyrosine (NT) and MDA-/HNE-protein adducts show good correlation with SLE disease activity [7], [9], [11], [15].

Reactive nitrogen species (RNS) are nitrogen-containing oxidants, i.e., nitric oxide (NO), peroxynitrite (ONOO**^−^**) and nitroxyl anion (HNO**^−^**) [16]. NO, generated by the enzyme inducible nitric oxide synthase (iNOS), is one of the most important and widely studied RNS. The potential of NO in disease pathogenesis lies largely to the extent of its production and generation of O_2_ **^.-^,** leading to formation of peroxynitrite (ONOO^-^). ONOO^-^ is a potent oxidizing agent which can react with tyrosine residues to form NT [4], [7], [12], [17], [18]. In addition, ONOO^−^-mediated modifications of endogenous proteins and DNA may enhance their immunogenicity, leading to a break in immune tolerance [6], [7], [19], [20]. Accumulating evidence in murine lupus shows an association between increasing iNOS activity and development and progression of ADs. Furthermore, studies using competitive inhibitors suggest that iNOS could play a pathogenic role in murine ADs [4], [17], [18], [21]. Growing observational data in humans also suggest that overexpression of iNOS and increased production of ONOO^−^ may contribute to glomerular and vascular pathology and to the pathogenesis of many other ADs [13], [20], [22]. Although there is appreciable evidence that NT, the marker of nitrosative modification of proteins, is enhanced in SLE and other ADs, and may contribute to the pathogenesis of these diseases [9], [19], [23], the potential mechanisms by which RNS contributes to the pathogeneses of ADs remain largely unexplored.

Trichloroethene (TCE), a common environmental contaminant and a widely used industrial solvent, has been involved in the development of ADs including SLE, systemic sclerosis and fascitis, both in human and animal studies [3], [8], [24]–[27]. Previous studies from our laboratory suggest that oxidative/nitrosative stress may contribute to TCE-induced autoimmunity [3],[21],[25],[27]. N-acetylcysteine (NAC), a precursor of intracellular glutathione, is known to provide cellular defense against oxidative stress [28]–[32]. Increasing lines of evidence suggest that NAC can also protect against nitrosative stress both in human and animals [33]–[37]. Earlier studies in our laboratory have demonstrated that NAC supplementation protected against autoimmunity by attenuating oxidative stress in MRL+/+ mice [32]. However, whether NAC could also protect against TCE-induced nitrosative stress remains unexplored. Therefore, the objectives of this study were: 1) To further evaluate the role of nitrosative stress in TCE-mediated autoimmune response, 2) to identify proteins susceptible to nitration, and 3) to assess the potential mechanisms by which NAC supplementation could provide protection against TCE-mediated autoimmunity. In order to achieve it, we treated MRL+/+ mice with TCE, NAC or TCE+NAC and examined the markers of autoimmunity and nitrosative stress in the sera and livers.

## Materials and Methods

### Animals and treatments

Five-week old female MRL+/+ mice (23–26 g) were purchased from The Jackson Laboratory (Bar Harbor, ME) and housed at the UTMB animal house facility maintained at ∼22°C, 50–60% relative humidity, and a 12 hr light/dark cycle. The animals were provided standard lab chow and drinking water ad libitum and were acclimated for 1 week prior to the treatment. The experiments were performed in accordance with the guidelines of the National Institutes of Health and were approved by the Institutional Animal Care and Use Committee of University of Texas Medical Branch. The mice, divided into 4 groups of 6 each, were treated with TCE, NAC or TCE plus NAC (TCE, 10 mmol/kg, i.p., every 4^th^ day; NAC, 250 mg/kg/day through drinking water) [3], [21], [25],[32],. The control mice received an equal volume of corn oil only. After 6 weeks of treatment, the animals were euthanized under nembutal (sodium pentobarbital) anesthesia, and blood was withdrawn from the inferior vena cava. Individual sera, obtained following blood clotting and centrifugation, were stored in small aliquots at −80°C until further analysis. At the same time, major organs were immediately removed and weighed. Portions of livers and kidneys from control and TCE-treated mice were snap-frozen in liquid nitrogen and stored at −80°C for the further analysis.

### Measurement of glutathione in the sera and livers

Glutathione (GSH) levels in the sera and livers were measured by using the Glutathione Assay kit (Cayman Chemical Co., Ann Arbor, MI) as per manufacturer's instructions. Briefly, liver homogenates (20%) were made in ice-cold phosphate-buffered saline (PBS; pH 7.4) containing 1 mM EDTA. A small amount of serum or liver supernatant (after centrifugation) was used for the protein assay (protein assay; Bio-Rad Laboratories). For deproteinization of sample, an equal volume of 10% w/v of metaphosphoric acid was added to the serum or residual supernatant. After centrifugation (10,000 *g*, 15 min), the resulting serum or liver supernatant was neutralized with 4 M of triethanolamine (50 µl/ml serum or supernatant) for the measurement of GSH levels in the samples. GSH concentration was then determined by the kinetic method as per the assay kit and was expressed as nmol mg^−1^ protein.

### Anti-nuclear and anti-histone antibodies in the serum

Serum levels of anti-nuclear antibodies (ANA) and anti-histone antibodies (AHA) were determined by using mouse-specific ELISA kits (Alpha Diagnostic Int'l, San Antonio, TX) as described earlier [8], [25], [27], [39].

### Quantification of nitrotyrosine and iNOS in serum

Nitrotyrosine (NT) levels in the mouse serum was quantitated by an ELISA kit (Cell Sciences, Norwood, MA), whereas iNOS was measured using an ELISA established in our laboratory earlier [21], [27].

### Quantitation of NT in the livers

For the quantitation of nitrated proteins in the livers of control, TCE-treated or TCE+NAC-treated mice, liver homogenates (10%, w/v) were made in PBS (pH 7.4) containing protease inhibitor cocktail (Sigma). The homogenates were centrifuged at 10,000 g at 4°C for 15 min and NT was quantitated in the supernatants by an ELISA (Cell Sciences) [21], [27].

### Western blot detection of iNOS in the livers

iNOS in the livers of MRL +/+ mice was also detected by Western blot analysis as described in our previous study [27]. Briefly, liver proteins from control, TCE-treated or TCE+NAC-treated mice were obtained using a lysis buffer (Pierce, Rockford, IL), and protein concentration in the lysates was determined by Bio-Rad Protein Assay reagent (Bio-Rad Laboratories, Inc., Hercules, CA). Fifty µg of protein dissolved in sample buffer was loaded onto a 12% Novex Tris-Glycine Gel (Invitrogen, Carlsbad, CA), resolved by electrophoresis, and subsequently transfered to nitrocellulose membrane. The membrane was incubated with TBS with 0.1% Tween-20 and 5% non-fat dry milk at room temperature for 2 h and subsequently probed with rabbit polyclonal anti-iNOS antibody for 2 h. Blots were washed thoroughly and incubated with horseradish peroxidase-conjugated goat anti-rabbit antibody (Upstate) for 1 h. iNOS bands were detected by using enhanced chemiluminescence (ECL) system (Amersham, Piscataway, NJ). The density of iNOS bands was analyzed with Eagle Eye II software (Stratagene, La Jolla, CA).

### RNA isolation and real-time PCR analysis for iNOS gene expression in liver

RNA isolation. RNA was isolated as described in our earlier studies [21], [32], [40]. Briefly, total RNA was isolated from livers using RiboPure kit (Ambion, Austin, TX). To eliminate contaminating genomic DNA, RNA preparation was treated with RNase free DNase I (DNA-free kit, Ambion, Austin, TX). The total RNA concentration was determined by measuring the absorbance at 260 nm. RNA integrity was verified electrophoretically by ethidium bromide staining and by measuring A260/A280 ratio.

Real-time PCR. The real-time PCR was performed as described earlier [21], [40]. Briefly, cDNA was prepared from isolated RNA by using SuperScript III First-Strand Synthesis Kit (Invitrogen, Carisbad, CA) described earlier [21]. Quantitative real-time PCR employing a two-step cycling protocol (denaturation and annealing/extension) was carried out using the primers (forward 5′-TGTCTGCAGCACTTGGATCA and reverse 5′-AACTTCGGAAGGGAGCAATG) by the Smart Cycler System. For each cDNA sample, parallel reactions were performed in triplicate for the detection of mouse iNOS and 18S. The reaction samples in a final volume of 25 µl contained 2 µl of cDNA templates, 2 µl primer pair, 12.5 µl iQ SYBR Green Supermix and 8.5 µl water. Amplification conditions were identical for all reactions: 95°C for 2 min for template denaturation and hot start prior to PCR cycling. A typical cycling protocol consisted of three stages: 15 s at 95°C for denaturation, 30 s at 65°C for annealing, 30 s at 72°C for extension, and an additional 6 s hold for fluorescent signal acquisition. To avoid the non-specific signal from primer-dimers, the fluorescence signal was detected 2°C below the melting temperature (T_m_) of individual amplicon and above the T_m_ of the primer-dimers [21], [32], [40]. A total of 45 cycles were performed for the studies.

Quantitation of PCR was done using the comparative *C*_T_ method as described in User Bulletin No. 2 of Applied Biosystems (Foster City, CA), and reported as fold difference relative to the calibrator cDNA (QuantumRNA Universal 18S Standards, Ambion). The fold change in iNOS cDNA (target gene) relative to the 18S endogenous control was determined by: 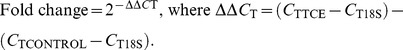



### Detection of nitrated proteins by 2D Gel and Western blot

The liver proteins from control, TCE-treated or TCE+NAC-treated mice were extracted as described previously [41]. Three hundred µg of total protein was added in 200 µl of 2-D protein extraction buffer-III containing 100 mM DTT, trace amount of bromophenol blue and 1% (v/v) IPG buffer pH 3–11 NL, and incubated at 21°C for 1 h. The proteins were then rehydrated to the DryStrip (11 cm, pH 3–11 NL) overnight at the same temperature. For each sample, IPG strips were used in duplicate. For the first dimension, isoelectric focusing (IEF) was performed at 20°C on EttanIPGphor3 (GE Healthcare, Sweden) in the following steps: 200 V for 30 min, 500 V for 1 h, 1000 V for 1.5 h, 8000 V for 2.5 h and 8000 V for 24,000 Vh. The strips were then equilibrated for 1 h in equilibration buffer (Tris–HCl 50 mM pH 8.8, urea 6M, DTT 100 mM, SDS 2%, and glycerol 20%). After rinsing two times with SDS-PAGE running buffer, the strips were loaded on to 10–20% SDS–tris–glycine gradient gel (13.3×8.7 cm) and were then run at 150 V for 2 h at room temperature in the 2nd dimension. Following the electrophoresis, one of the duplicate gels of each sample was stained with Coomassie blue G250 (CBB G250), while the other one was used for Western blotting by transferring the proteins to PVDF membrane as described previously (Benndorf and Babel, 2002). The PVDF membrane was blocked with 5% fat-free milk in TBST (pH 7.4) for 1 h at room temperature and then incubated with anti-3NT IgG (1∶4000 dilution in TBST containing 5% fat-free milk, pH 7.4) at 4°C overnight. The membrane was washed and incubated with HRP conjugated secondary antibody (1∶8000 dilution in TBST containing 5% fat-free milk) for 45 min at room temperature. The signal was visualized by enhanced chemiluminescent detection.

### SameSpots analysis of protein expression variation

2D gel images (CBB G250 stained) were acquired by 2D Proteomic Imaging System (ProXPRESS, PerkinElmer, Inc.). The scanning resolution of CCD camera (software, ProScan V4.0.0.10) was 100 µm with white light and the exposure time is 600 ms. The 2D gel images were then analyzed by the software Progenesis SameSpots (nonlinear dynamics, version 4.0) which has been judged to be much improved in reproducibility and objectivity compared to previous generations of 2D gel analysis software (Silva et al., 2010). To match the same spot (same protein) between overlapped gel images, one of the six gels was chosen as reference. Spot volumes were normalized to those of the reference gel to obtain normalized volumes that are comparable across gels. Protein expression fold changes between controls and aniline-treated rats were determined as described below in the statistical analysis.

### Trypsin digestion and MALDI TOF/TOF MS analysis

The nitrated protein spots were manually picked up from the 2D gel. The protein was digested with trypsin (0.1 µg per spot, Promega) in 10 µl of 25 mM ammonium bicarbonate, pH 8.0, for 6 h at 37°C. One µl of digested sample solution was used for MALDI TOF/TOF MS. The data was collected by using an Applied Biosystems 4800 MALDI TOF/TOF proteomics analyzer. The instrument was operated in a positive ion reflection mode with mass range from 850 to 3000 Da. The focus mass was set at 1700 Da. For MS data, 2000–4000 laser shots were acquired and averaged from each sample spot. Following MALDI MS analysis, MALDI MS/MS was performed on several (5–10) abundant ions from each sample spot. A 1 kV positive ion MS/MS method was used to acquire data under post-source decay (PSD) conditions. The instrument precursor selection window was ±3 Da. For MS/MS data, 2000 laser shots were acquired and averaged from each sample spot. Automatic external calibration was performed using reference fragment masses 175.120, 480.257, 684.347, 1056.475, and 1441.635 (from precursor mass 1570.700).

### Proteins identification

Applied Biosystems GPS Explorer™ (Version 3.6) software was employed for searching the respective protein database using both MS and MS/MS spectral data for protein identification. Protein match probabilities were determined by using MASCOT scores, and a score of more than 61 was considered significant (p<0.05). MS peak filtering included the following parameters: mass range 800 Da to 4000 Da, minimum S/N filter  = 10, mass exclusion list tolerance  = 0.5 Da, and mass exclusion list (for some trypsin and keratin-containing compounds) included masses 842.51, 870.45, 1045.56, 1179.60, 1277.71, 1475.79, and 2211.1. For MS/MS peak filtering, the minimum S/N filter  = 10. The mass data was matched to the NCBI protein database. Precursor tolerance was set at 0.2 Da; MS/MS fragment tolerance was set at 0.3 Da; mass  =  monoisotopic; and peptide charges were only considered as +1.

### Western blot analysis for NF-κB p65

Western blot analysis was done to analyze the expression and activation of NF-κB p65 in the livers. Briefly, liver lysates were prepared by using the lysis buffer essentially as described by the manufacturer (Cell Signaling, Beverly, MA). The lysate proteins were subjected to 10% SDS-PAGE and transferred to a PVDF membrane (Amersham, Arlington Heights, IL). After blocking with non-fat dry milk (5%, w/v), the membrane was incubated with antibodies specific for NF-κB p65 and phosphor-NF-κB p65 (p-NF-κB p65; Cell signaling). The rest of the procedure was the same as described previously [40].

### Statistical analyses

The values are means ± SD. One-way ANOVA followed by Tukey-Kramer multiple comparisons test (GraphPad Instat 3 software, La Jolla, CA) was performed for the statistical analysis. A *p* value <0.05 was considered to be statistically significant.

## Results

### Effect of TCE on GSH in the sera and livers

**Figure 1:**
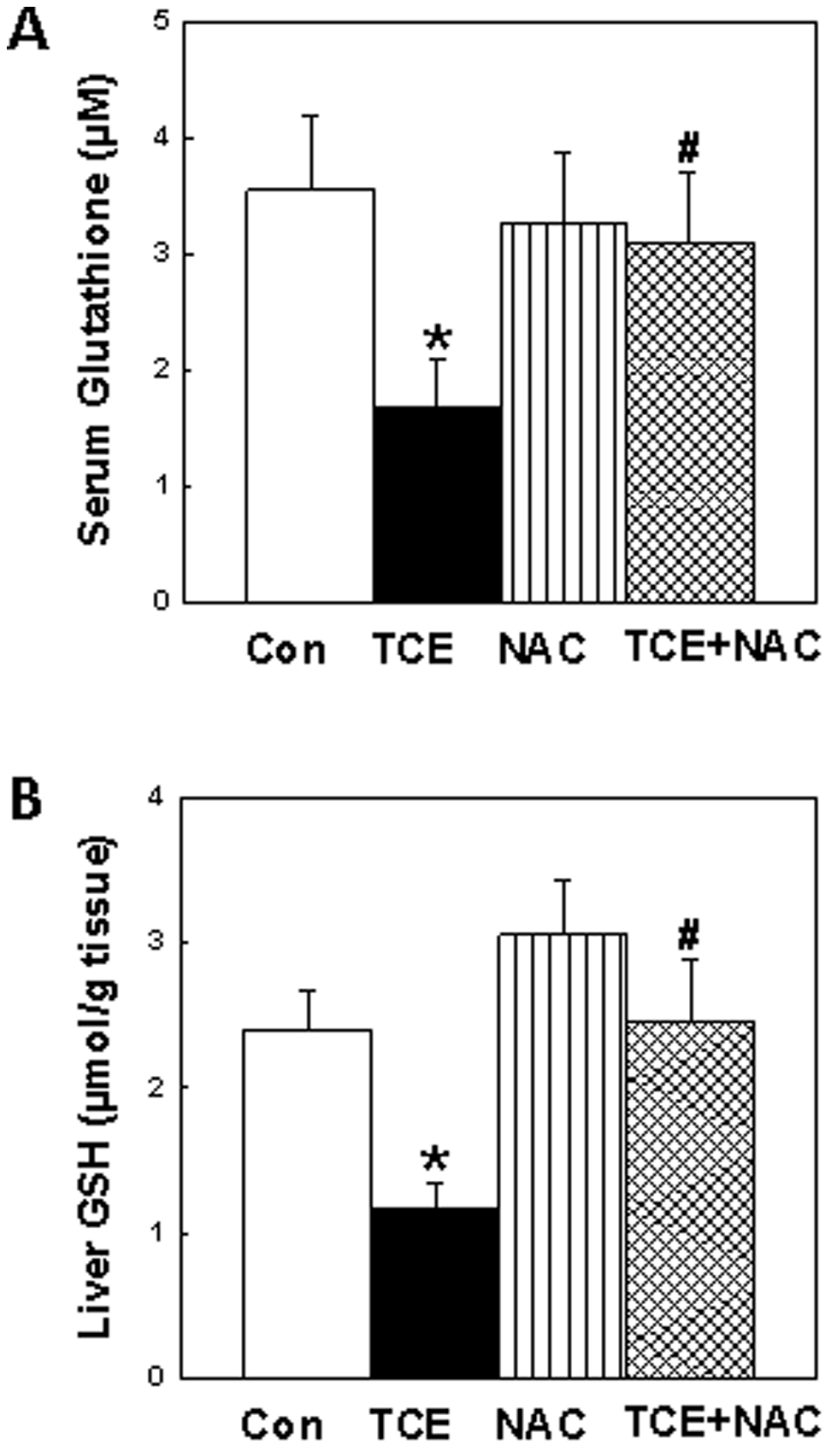
The levels of GSH in the sera (A) or livers (B) of MRL+/+ mice treated with TCE, NAC or TCE+NAC for 6 weeks. The values are means ± SD. *p<0.05 vs. controls; ^#^p<0.05 vs. TCE-treated mice.

Glutathione (GSH), an endogenous antioxidant, prevents damage to cellular components caused by reactive oxygen species [28], [42], [43]. TCE is known to generate free radicals [3], [8], [27], [44], [45] and NAC is a precursor of GSH. To assess the redox status following TCE, NAC or TCE+NAC exposure and evaluate the contribution of ROS/RNS in TCE-induced autoimmunity, the glutathione level was quantitated in the sera and livers. As evident from Fig. 1, TCE exposure caused significant decreases in GSH levels both in the sera and livers as compared to the controls. However, NAC supplementation attenuated the GSH levels as evident from significantly increased GSH levels in mice treated with TCE+NAC as compared to TCE only group.

### Serum autoantibodies in MRL+/+ mice

Autoantibodies, such as ANA and AHA, are considered important indices and biomarkers of ADs [46], [47]. These autoantibodies were analyzed in the sera of MRL+/+ mice treated with TCE, NAC or TCE+NAC (Table 1). In comparison to controls, there were significant increases in serum ANA and AHA levels in mice treated with TCE. NAC supplementation, however, significantly attenuated their levels, providing support that NAC supplementation improves or averts the autoimmunity mediated by TCE.

**Table 1:** Serum autoantibodies in the mice treated with TCE, NAC or TCE+NAC.

	ANA ( µg/ml)	Anti-histone antibodies (OD at 450 nm)
Controls	105.65±16.21	0.348±0.050
TCE	171.32±31.23*	0.497±0.096*
NAC	96.51±14.15	0.325±0.062
TCE+NAC	116.29±19.92^#^	0.372±0.071^#^

The values are means ± SD. * p < 0.05 vs. controls; ^#^ p<0.05 vs. TCE-treated mice.

### Nitrotyrosine levels in the serum and livers

**Figure 2:**
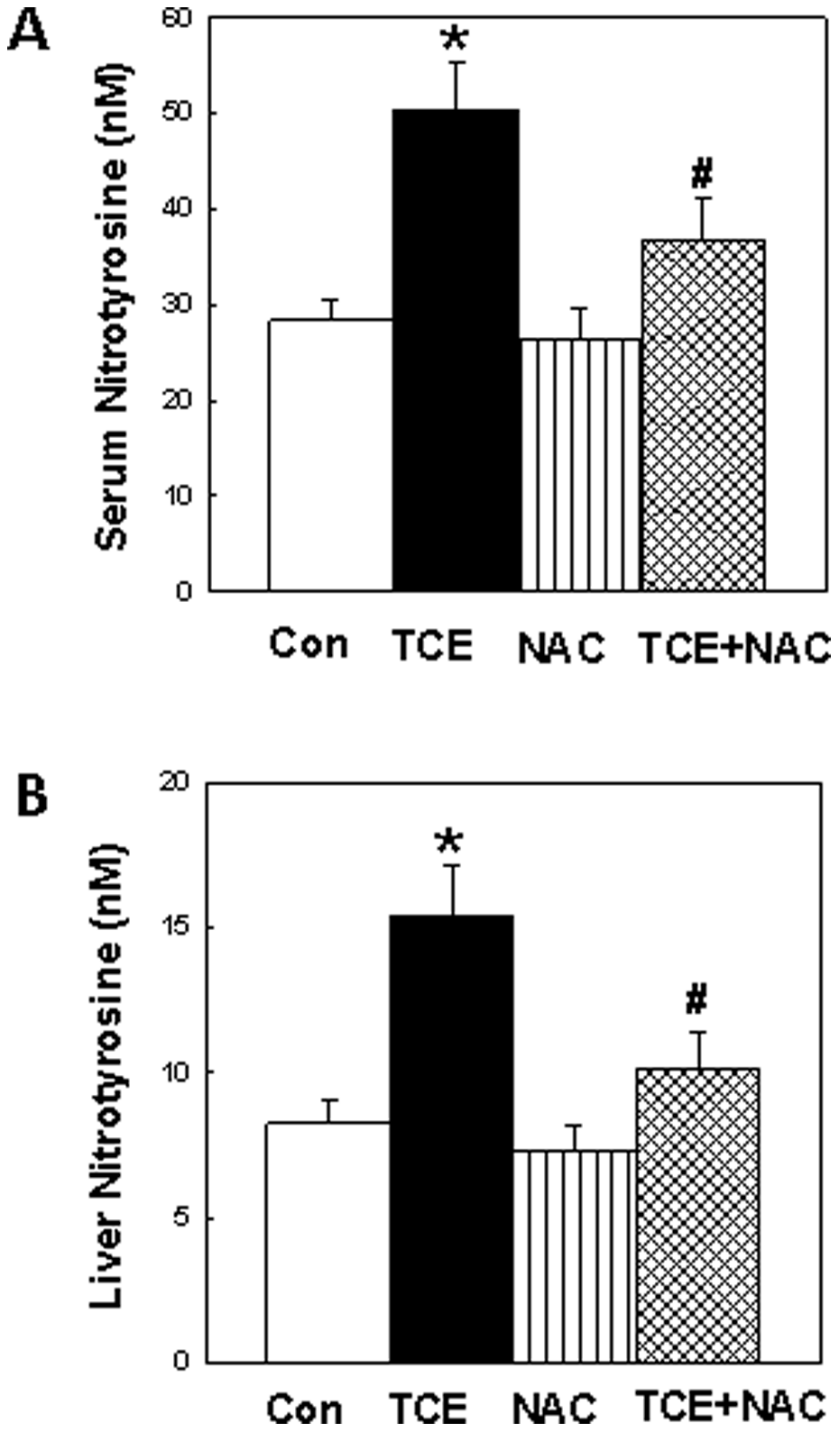
The formation of nitrotyrosine in the sera (A) or livers (B) of MRL+/+ mice treated with TCE, NAC or TCE+NAC for 6 weeks. The values are means ± SD. *p<0.05 vs. controls; ^#^p<0.05 vs. TCE-treated mice.

NT formation is considered to be a biomarker of RNS production [4], [9], [19]. To assess the involvement of nitrosative stress in TCE-mediated autoimmune response, we determined the serum levels of NT in control and TCE, NAC or TCE+NAC-treated mice. Fig. 2 shows that TCE exposure led to significant increases in serum NT levels, which were attenuated by NAC supplementation (Fig. 2A). The NT levels in liver, a major organ where TCE is known to generate free radicals and lead to autoimmune damages [26], [39], [44], [48], were also analyzed. The NT levels in livers were also significantly higher in TCE-treated mice in comparison to the controls and NAC supplementation attenuated the increases in NT (Fig. 2B).

### iNOS in the sera and livers

**Figure 3:**
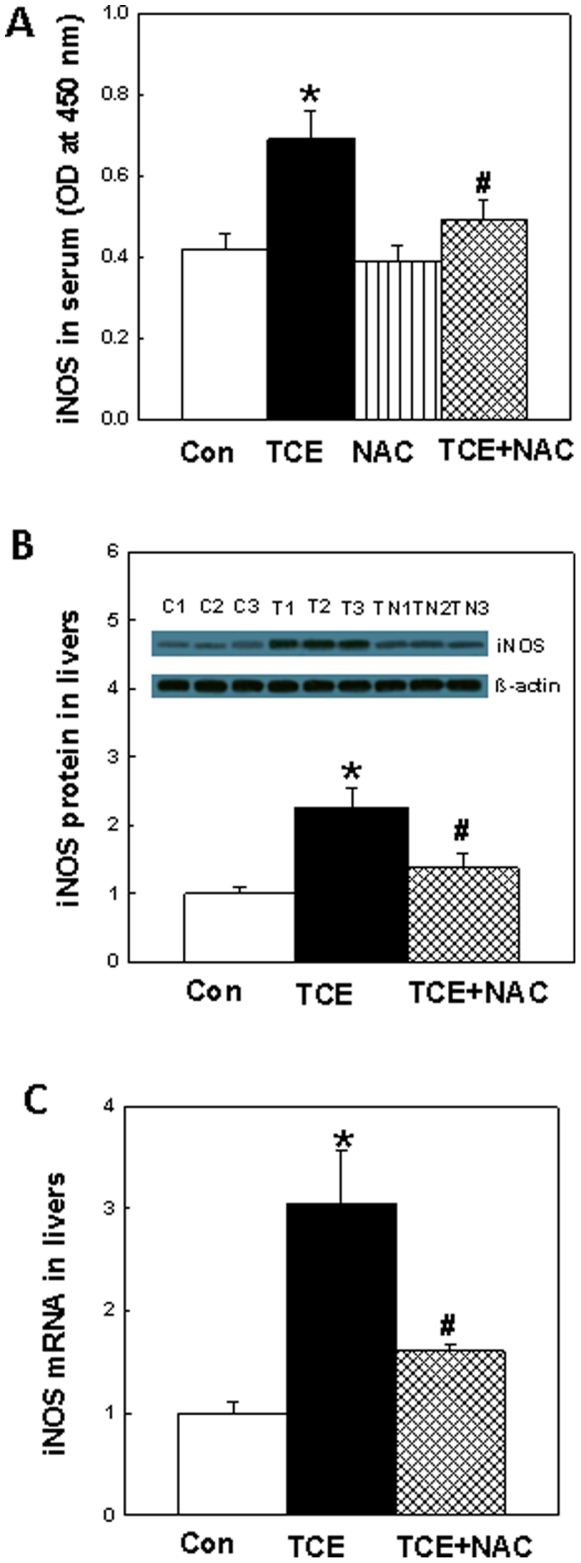
iNOS protein and mRNA expression in the sera (A) or livers (B and C) of MRL+/+ mice treated with TCE, NAC or TCE+NAC for 6 weeks. The values are means ± SD. *p<0.05 vs. controls; ^#^p<0.05 vs. TCE-treated mice. β-actin was used as loading control.

iNOS catalyzes the formation of NO - the most important RNS [16]. iNOS levels in sera, quantitated by specific ELISA, in control, TCE, NAC or TCE+NAC are presented in Fig. 3A. The levels of iNOS in TCE-treated mice was significantly increased in comparison to the controls, but the increases were attenuated by NAC supplementation. The iNOS protein expression in the livers was also determined by Western blot analysis. The results show that iNOS expression increased significantly in the livers of TCE-treated mice (2.4 folds, Fig. 3B) compared to the controls, and the increases in iNOS expression were attenuated by the NAC supplementation.

To determine the impact of TCE exposure on iNOS regulation, the iNOS mRNA expression was analyzed using real-time PCR in the livers of mice treated with TCE, NAC or TCE+NAC. The mRNA levels in livers of TCE-treated mice increased significantly (3.1-folds) in comparison to the controls (Fig. 3C). Interestingly, NAC supplement also attenuated the increases in mRNA. The changes in liver mRNA expression matched well with protein expression increases as determined by Western blot (Fig. 3B).

### Expression and activation of NF-κB p65 in the livers

**Figure 4:**
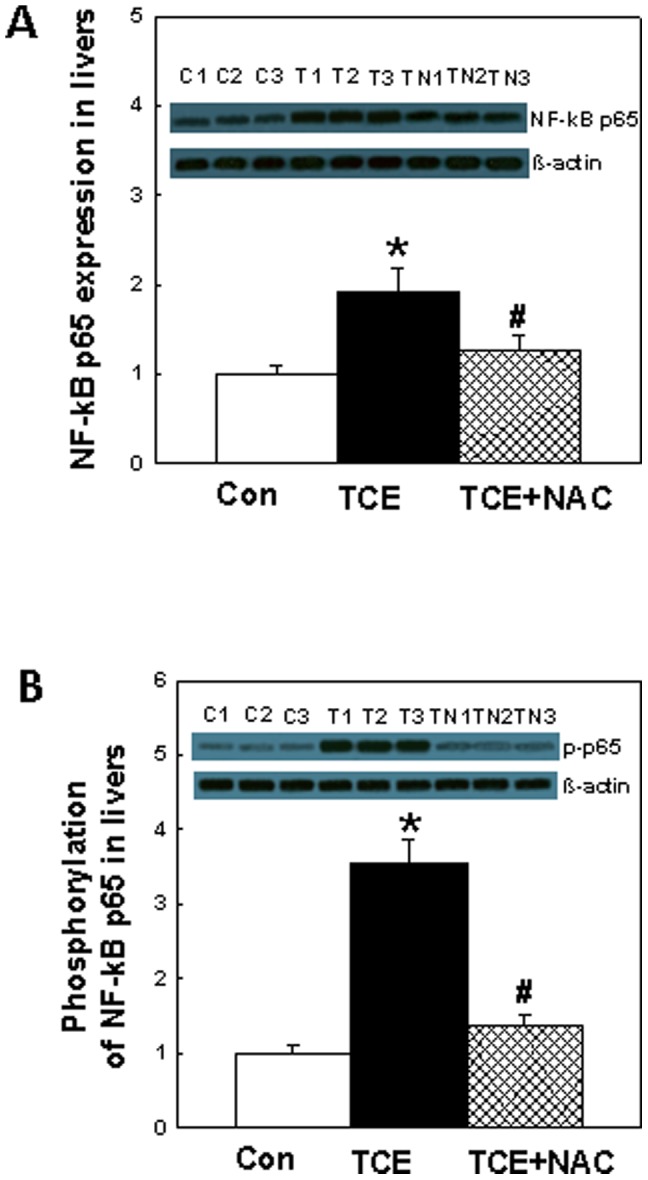
Protein expression (A) and phosphorylation (B) of NF-κB p65 in the livers of MRL+/+ mice treated with TCE, NAC or TCE+NAC for 6 weeks. The values are means ± SD. *p<0.05 vs. controls; ^#^p<0.05 vs. TCE-treated mice. β-actin was used as loading control.

NF-κB has been shown to be involved in a number of ADs as a critical regulator of a variety of pro-inflammatory genes. Recent reports demonstrated that NF-κB may regulate iNOS expression and NO production [49]–[51]. NAC has been shown to modulate inflammatory responses through signaling pathways that control pro-inflammatory NF-κB activation [29], [52]. Since the results of this study show significantly increased iNOS protein and mRNA expression following TCE exposure, it was, therefore, of interest to analyze NF-κB activation. NF-κB p65 expression in livers was determined by Western blot analysis. Fig. 4A shows a significant increase of ∼1.9 fold in NF-κB p65 levels in TCE-treated mice in comparison to the controls, and their attenuation following NAC supplementation. To further evaluate the activation of NF-κB p65, phosphorylation of NF-κB p65 in the livers was analyzed as shown in Fig. 4B. The p-NF-κB p65 was remarkably elevated (3.6 fold) in the livers of TCE-treated mice and attenuated following NAC supplementation.

### Nitrated protein spots identified by 2D Western blotting

**Figure 5:**
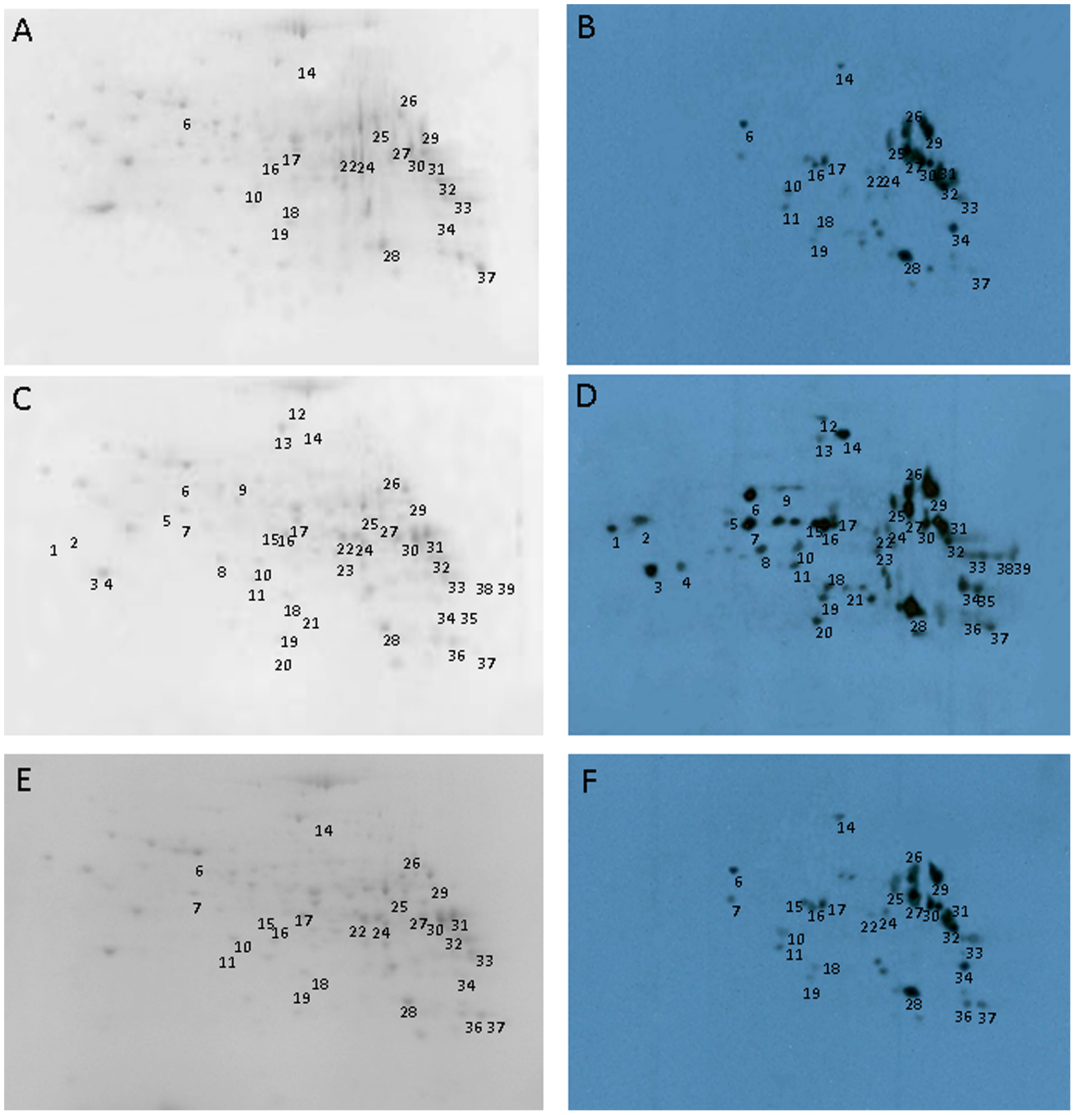
2D gel profile of proteins in livers of control (A, B), TCE-treated (C, D) or TCE+NAC-treated (E, F) MRL+/+ mice. The protein spots for nitrated proteins, identified by western blot (B, D and F), also matched to the 2D gel protein profile in (A, C and F - CBB G250 stained). The numbers used for the spots are the same as in the Tables.

Since our ELISA results showed increased NT in the livers following TCE exposure, it was of interest to identify the nitrated proteins. To achieve that, 2D gels for each sample were run in duplicate, one for CBB G250 stain and the other one for Western blot analysis. The 2D gel protein profile of a representative control sample is shown in Fig. 5A, and Fig. 5B is the corresponding Western blot map of nitrated proteins of the same sample. The nitrated proteins shown in black circles (Fig. 5B) were matched to the 2D gel protein profiles in Fig. 5A. Similarly, the 2D gel protein profiles of liver extract from a TCE-treated or a TCE+NAC-treated mouse is shown in Fig. 5C and Fig. 5E, whereas Fig. 5D and Fig. 5F show the corresponding Western blot map of nitrated protein spots of the same samples. The black circled spots (Fig. 5D and Fig. 5F) were also matched to the 2D gel protein profiles in Fig. 5C and Fig. 5E. As shown in the figures, the nitrated proteins spots were found in the pI range of pH 4.9 to 8.8 and molecular weights of 19.1 to 128.3 kDa. The 2D gel protein profiles of randomly chosen three controls, three TCE-treated and three TCE+NAC-treated mice matched well when analyzed by using SameSpots program (data not shown). Among the three controls, we identified 21 nitrated protein spots which were also present in TCE-treated protein extracts. The protein extracts from TCE-treated mice showed remarkably greater protein nitration, which was evident from increased number of spots for nitrated proteins. We identified a total of 39 nitrated protein spots among the three TCE-treated samples, out of which 18 were found only in the TCE-treated protein extracts (Fig. 5D). Interestingly, we only identified a total of 24 nitrated protein spots among the three TCE+NAC-treated samples, which were also observed in the TCE-treated protein extracts (Fig. 5F). These nitrated protein spots (same protein spots present in at least two samples) were picked up and subjected to MALDI TOF/TOF MS/MS analyses.

### Identified nitrated proteins in the liver

Combination of MALDI TOF/TOF MS and MS/MS and protein database search enabled us to further identify the nitrated proteins. Probability-based MASCOT score was used to evaluate the identifications. The MASCOT score of more than 61 represents the statistical confidence >95% (p<0.05). A total of 31 nitrated proteins were identified (MASCOT score>61; Tables 2, 3), of which 12 were found in TCE treated liver extracts only (Table 3), 2 were found in both TCE-treated and TCE+NAC-treated mice (Table 3), and 17 were found in all of the 3 groups (Table 2). The major nitrated proteins following TCE treatment included skeletal proteins like beta-actin (No. 8); enzymes such as mitochondrial ATP synthase (No. 1), methionine adenosyltransferase I (Nos.3, 4), carbamoyl-phosphate synthetase 1 (No. 12), 3-hydroxyanthranilate 3,4-dioxygenase (No. 20), glutathione transferase (No. 36); stress proteins and chaperones like heat shock protein 1 (No. 6), respectively (Tables 2 and 3).

**Table 2:** Summary of nitrated proteins in control, TCE- and TCE+NAC-treated mice.

No.*	Protein name	Accession No.^#^	MW(Da)	Peptide No	Score^+^	Score C.I.%	Protein PI
6	heat shock protein 1 (chaperonin)	gi|183396771	61088.4	7	255	100	5.91
10	fructose-1,6-bisphosphatase 1	gi|9506589	37287.9	8	465	100	6.15
11	peroxiredoxin 6	gi|6671549	24925	14	676	100	5.98
14	Chain A, Crystal Structure Of Fumarylacetoacetate	gi|13399972	46560.2	6	134	100	6.99
16	sorbitol dehydrogenase precursor	gi|1009706	40635.8	7	362	100	6.6
17	sorbitol dehydrogenase precursor	gi|1009706	40635.8	10	390	100	6.6
18	fructose-1,6-bisphosphatase 1	gi|9506589	37287.9	10	409	100	6.15
19	Arg1	gi|71059675	34927.2	11	568	100	6.51
22	malate dehydrogenase 1, NAD (soluble), isoform	gi|148675904	40319.1	6	346	100	7.07
24	malate dehydrogenase 1, NAD (soluble), isoform	gi|148675904	40319.1	6	346	100	7.07
25	sorbitol dehydrogenase, isoform CRA_b	gi|148696143	38794.9	9	284	100	7.01
26	glutamate dehydrogenase 1	gi|148692928	54526.7	15	399	100	7.66
27	glutamate dehydrogenase 1	gi|148692928	54526.7	14	396	100	7.66
28	peroxiredoxin 1	gi|6754976	22390.4	7	110	100	8.26
29	mutant catalase	gi|15004258	59985.7	10	441	100	7.72
30	mCG15755	gi|148700170	46840.1	11	179	100	8.36
31	mCG15755	gi|148700170	46840.1	11	179	100	8.36
32	carbonic anhydrase 3, isoform CRA_b	gi|148673185	31540.8	12	347	100	8.43
33	acetyl-Coenzyme A acyltransferase 2 (mitochondrial)	gi|148677565	38711.8	10	421	100	8.59
34	arylsulfotransferase ST1A4	gi|5420463	34922.6	6	156	100	8.18
37	aldolase 2, B isoform, isoform CRA_d	gi|148670367	19164.1	6	102	100	8.91

*Spot numbers are the same as shown in the Fig.5; ^#^The accession number are in NCBI database; ^+^A score >64 was considered significant (p<0.05) for protein identified from the database.

**Table 3:** Summary of nitrated proteins in TCE- or TCE+NAC-treated mice.

No.*	Protein name	Accession No.^#^	MW(Da)	Peptide No	Score^+^	Score C.I.%	Protein PI
1	mitochondrial ATP synthase, H+ transporting F1	gi|89574015	48047	15	721	100	4.9
2	alpha-fetoprotein	gi|191765	48791.6	6	141	100	5.47
3	methionine adenosyltransferase I, alpha	gi|19526790	44051.2	7	244	100	5.51
4	methionine adenosyltransferase I, alpha	gi|19526790	44051.2	7	244	100	5.51
5	selenium binding protein 1	gi|22164798	53050.6	9	615	100	5.87
7@	selenium binding protein 1	gi|22164798	53050.6	9	615	100	5.87
8	put. beta-actin (aa 27–375)	gi|49868	39445.8	14	604	100	5.78
9	mCG114361	gi|148682321	48067.4	9	225	100	6.08
12	carbamoyl-phosphate synthetase 1	gi|187466221	128298.6	10	142	100	6.22
13	sarcosine dehydrogenase precursor	gi|20149748	102644.3	20	650	100	6.28
15@	sorbitol dehydrogenase precursor	gi|1009706	40635.8	8	242	100	6.6
20	3-hydroxyanthranilate 3,4-dioxygenase	gi|17921976	32954.6	14	266	100	6.09
21	nit protein 2	gi|12963555	30824.7	9	359	100	6.44
23	F1 protein	gi|1841443	43784.3	9	533	100	6.57
35	PREDICTED: similar to glyceraldehyde-3-phosphate	gi|149258934	36074.3	8	206	100	8.44
36@	glutathione transferase	gi|193703	25401.3	8	203	100	8.76
38	mCG9091, isoform CRA_c	gi|148700301	34387.7	7	219	100	9.11
39	mCG9091, isoform CRA_c	gi|148700301	34387.7	8	135	100	9.11

All these nitrated proteins were identified in TCE-treated mice; ^@^Nitrated proteins also identified in TCE+NAC-treated mice; *Spot numbers are the same as shown in the Fig.5; ^#^The accession number are in NCBI database; ^+^A score >64 was considered significant (p<0.05) for protein identified from the database.

## Discussion

Several lines of evidence in autoimmune-prone MRL+/+ mice demonstrated that increased ROS/RNS generation was associated with increased formation of autoantibodies, suggesting a potential role of oxidative/nitrosative stress in TCE-mediated autoimmune response [3], [8], [9], [27], [39]. However, the molecular mechanisms have not been clearly elucidated. Previous studies in our laboratory showed that NAC attenuated TCE-mediated autoimmunity by providing protection against oxidative stress [32]. Increasing evidences in recent years support that NAC is also capable of suppressing iNOS expression and consequently diminishing nitrosative stress [10], [33]-[35], [37]. This study was, therefore, aimed to support our previous findings and provide new mechanistic evidence for the role of nitrosative stress in TCE-mediated autoimmune response by treating the female MRL +/+ mice and evaluating the markers of nitrosative stress for their association with the markers of autoimmune response.

NAC is a well-known antioxidant and the precursor of glutathione which plays a critical role on the immune system and can influence the disease outcome including SLE and RA [30], [32], [42]. Previous studies suggest that NAC improves redox status via decreasing oxidative/nitrosative stress both in humans and animals, and can even reduce disease activity in SLE patients [10], [28]-[33]. This study not only demonstrated that TCE exposure is associated with GSH depletion and autoimmune response as evident from significantly decreased levels of GSH and increased autoantibodies in TCE-exposed mice, but also showed that NAC supplementation averted GSH depletion and also provided protection against TCE-induced autoimmunity. More interestingly, the level of GSH showed good relationship with the autoimmune response, providing further support to earlier findings and suggesting that NAC has the potential to improve or avert TCE-induced autoimmune disorder.

Nitric oxide (NO), which is formed in excessive amounts as a result of iNOS activation, is considered to contribute to SLE and other ADs via reacting with superoxide to form ONOO^-^ [17], [18], [20]. Overexpression of iNOS is associated with the development and progression of ADs both in human and experimental animals, and studies using iNOS inhibitors suggest a pathogenic role of iNOS in murine ADs [4], [17], [18]. The increases in iNOS activity have also been shown to be associated with increased formation of NT [4], [17]. This led us to test the response of iNOS, NT formation and protein nitration and their potential contribution to TCE-mediated autoimmunity. Our results showed the increases in iNOS activity, protein and mRNA expression accompanied by enhanced formation of NT with more nitrated proteins in TCE-treated mice as compared to controls. Interestingly, NAC supplementation significantly suppressed the overexpression of iNOS and NT formation mediated by TCE exposure, and also protected a few important enzymes such as mitochondrial ATP synthase, methionine adenosyltransferase I, carbamoyl-phosphate synthetase 1, 3-hydroxyanthranilate 3,4-dioxygenase, glutathione transferase, skeletal proteins like beta-actin and stress proteins and chaperones like heat shock protein 1 from nitration, suggesting that NAC could significantly improve or avert TCE-induced nitroative stress. It is also apparent from our data that nitrosative stress is strongly associated with autoimmune response. These results apart from providing support to earlier findings that oxidative stress might play a role in TCE-mediated autoimmune response [21], [27], also provide new evidence for an association among NAC supplementation, nitrosative stress and the etiology of TCE-induced autoimmunity. To our knowledge, this is the first study to demonstrate that NAC provides protection against TCE-mediated autoimmunity by attenuating nitrosative stress.

**Figure 6:**
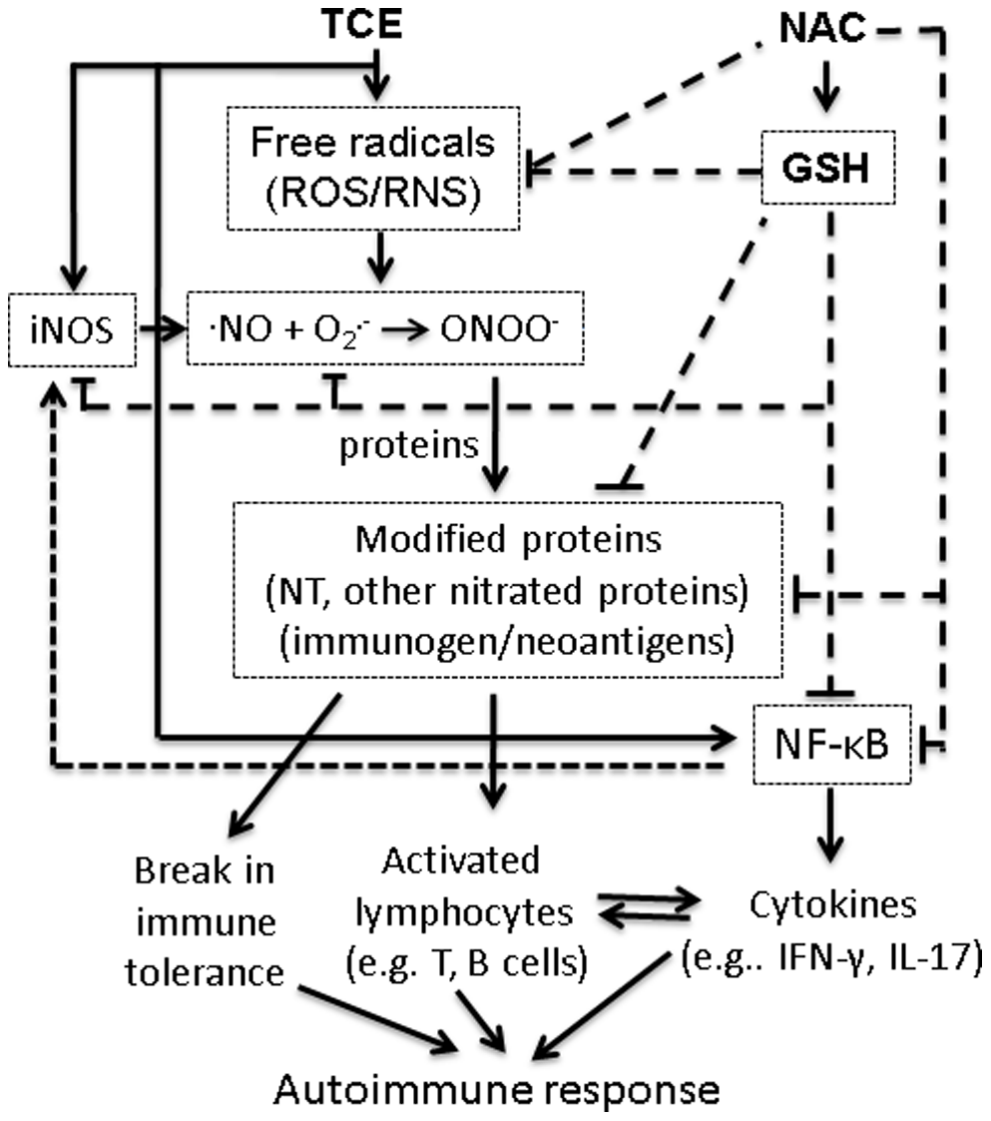
The plausible mechanisms of TCE-induced autoimmune response and its attenuation by NAC supplementation.

It is evident from our data that TCE exposure also resulted in an increased expression and activation of NF-κB p65. Interestingly, NAC supplementation not only ameliorated the TCE-induced nitrosative stress, GSH and NF-κB p65 activation, but also the markers of autoimmune response, as evident from decreased levels of autoantibodies in the sera. NAC treatment can reduce the formation of ONOO**^-^** possibly by a simultaneous inhibition of ROS and NO via the suppression of NF-κB-mediated induction of iNOS expression [29], [33], [53]. Based on these findings, we conclude that TCE exposure activated iNOS and generated free radicals (RONS) leading to increased ONOO**^-^**, which led to increased formation of modified proteins (NT or nitrated proteins) which can act as immunogen or neoantigens. These immunogen/neoantigens might activate lymphocytes or cause break in immune tolerance, leading to autoimmune response [6]-[8], [21]. NAC supplementation could ameliorate TCE-induced autoimmunity potentially via suppressing/averting NF-κB and iNOS activity or by directly scavenging free radicals (O_2_ **^.-^**, NO and ONOO**^-^**) leading to reduction in neoantigen formation and thus, an autoimmune response [29], [32], [33], [54], [55]. Fig. 6 depicts the potential pathways of TCE-induced autoimmunity and its attenuation following NAC supplementation. Our results thus, not only provide support to the role of nitrosative stress in TCE-induced autoimmune response, but also provide a map for further investigating alterations in these nitrated proteins' structural and functional properties, which could lead to a better understanding of the role of protein nitration in the pathogenesis of TCE-mediated autoimmunity. Attenuation of TCE-induced autoimmunity in mice by NAC could be important in developing preventive and/or therapeutic strategies.
